# Lateral Heel Pain Caused by Impingement of Hypertrophic Peroneal Tubercle and Os Peroneum

**DOI:** 10.1155/2021/6621539

**Published:** 2021-01-08

**Authors:** Ryosuke Takada, Song Ho Chang, Taro Kasai, Masashi Naito, Jun Hirose, Sakae Tanaka, Takumi Matsumoto

**Affiliations:** ^1^Department of Orthopedic Surgery, Faculty of Medicine, The University of Tokyo, 7-3-1 Hongo, Bunkyo-ku, Tokyo 113-8655, Japan; ^2^Department of Orthopedic Surgery, Teikyo University Hospital, Mizonokuchi, 5-1-1 Niko Takatsu-ku, Kawasaki City, Kanagawa 213-8507, Japan; ^3^Department of Orthopedic Surgery, Tokyo Metropolitan TAMA Medical Center, 2-8-29 Musashidai, Fuchu City, Tokyo 183-8524, Japan; ^4^Department of Orthopedic Surgery, Japan Community Health Care Organization, Tokyo Shinjuku Medical Center, 5-1 Tsukudo cho, Shinjuku-ku, Tokyo 162-8543, Japan

## Abstract

Hypertrophic peroneal tubercle (HPT) is an overgrowth of the peroneal tubercle located on the lateral aspect of the hindfoot, which could cause tenosynovitis of the peroneus longus tendon. Os peroneum (OP) is an accessory ossicle that exists in the peroneus longus tendon at the lateral aspect of the calcaneocuboid joint. Both HPT and OP can cause lateral foot pain and occasionally require surgical treatment. We encountered a case of lateral foot pain of HPT coexisting with OP. Careful preoperative magnetic resonance imaging, dynamic ultrasonographic image, and block injection suggested an impingement of HPT and OP as a cause of lateral foot pain. Surgical resection of HPT, while retaining OP, successfully achieved pain relief in the patient. To the best of our knowledge, this is the first report presenting a case of HPT coexisting with OP successfully treated without OP resection.

## 1. Introduction

Hypertrophic peroneal tubercle (HPT), an overgrowth of the peroneal tubercle, may cause a stenosing tenosynovitis of the peroneal tendon on the lateral hindfoot and require surgical treatment [1]. Os peroneum (OP) is a sesamoid bone existing inside the peroneus longus, which can cause painful os peroneum syndrome (POPS). POPS includes a variety of pathologies, such as fractures or diastases of multipartite OP and damage in the peroneus longus tendon, and requires surgery [2, 3]. There are a limited number of studies reporting the surgical treatment outcomes in cases involving both HPT and OP, and an optimal procedure for such a condition has not been established. We herein report a case of HPT coexisting with OP that was successfully treated with resection of HPT, but retaining OP.

## 2. Case Presentation

**Figure 1:**
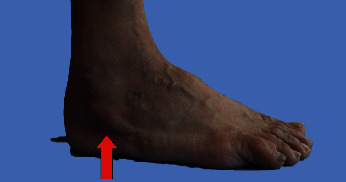
A bony protrusion had been palpitated on the lateral aspect of hindfoot (red arrow).

A 63-year-old man reported pain in the lateral aspect of his right hindfoot during ambulation for 6 years. During the initial examination, a bony protrusion was palpated on the lateral aspect of his right hindfoot (Figure 1). The patient complained of pain at the same site during walking.

**Figure 2:**
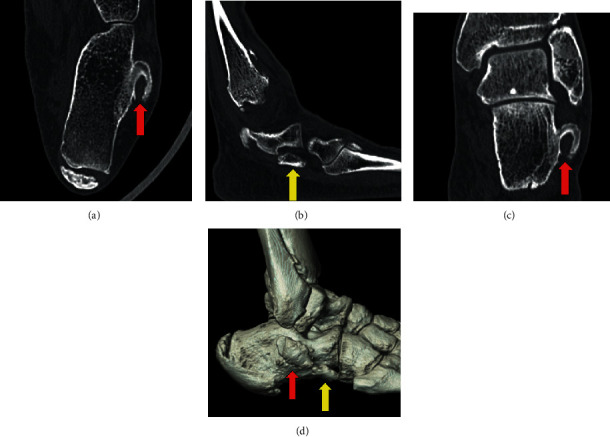
CT: Axial image (a), sagittal image (b), coronal image (c), and 3D image (d). Hypertrophic peroneal tubercle, a bony overgrowth surrounding a peroneal tendon (red arrow). Os peroneum, an ossicle on the outer anterior side of the calcaneus (yellow arrow).

**Figure 3:**
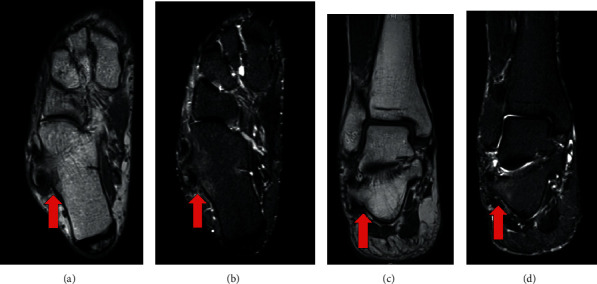
Right foot MRI: T1-weighted axial image (a), STIR axial image (b), T1-weighted coronal image (c), and STIR coronal image (d). Low-intensity lesion on T1-weighted image and high-intensity lesion on STIR image were detected around hypertrophic peroneal tubercle, and bone marrow lesions were suggested (red arrow).

**Figure 4:**
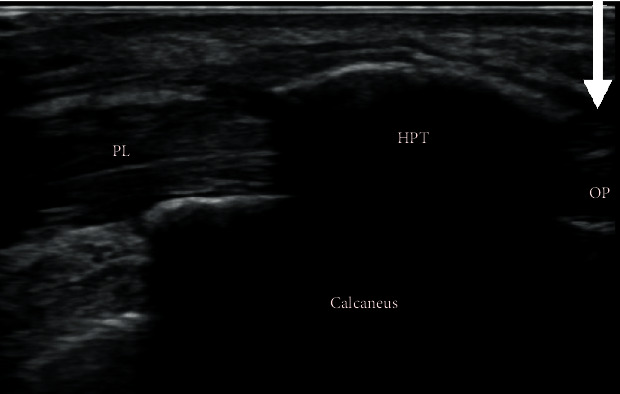
Dynamic ultrasonographic image confirmed hypertrophic peroneal tubercle and os peroneum causing impacts. Lidocaine injection was performed in between and os peroneum (white arrow). Abbreviation: PL: peroneus longus; HTP: hypertrophic peroneal tubercle; OP: os peroneum.

Plain radiographs and computed tomography (CT) images of his right foot showed HPT, bony overgrowth surrounding the peroneal tendon, and OP, an ossicle on the outer anterior side of the calcaneus (Figure 2). Magnetic resonance imaging (MRI) showed a low-intensity lesion on T1-weighted image and a high-intensity lesion on a short T1 inversion recovery (STIR) image around HPT (Figure 3). Bone marrow lesions of HPT were suggested, while OP did not show any signs of inflammation. Dynamic ultrasonographic images confirmed HPT and OP causing impacts accompanied with active eversion of the foot, and the pain was released by lidocaine injection between the HPT and OP (Figure 4). On the basis of these findings, we assumed that the lateral foot pain stemmed from inflammation around the HPT caused by the impacts between the HPT and OP and not from POPS. Therefore, we decided to perform only HPT resection, without OP removal.

**Figure 5:**
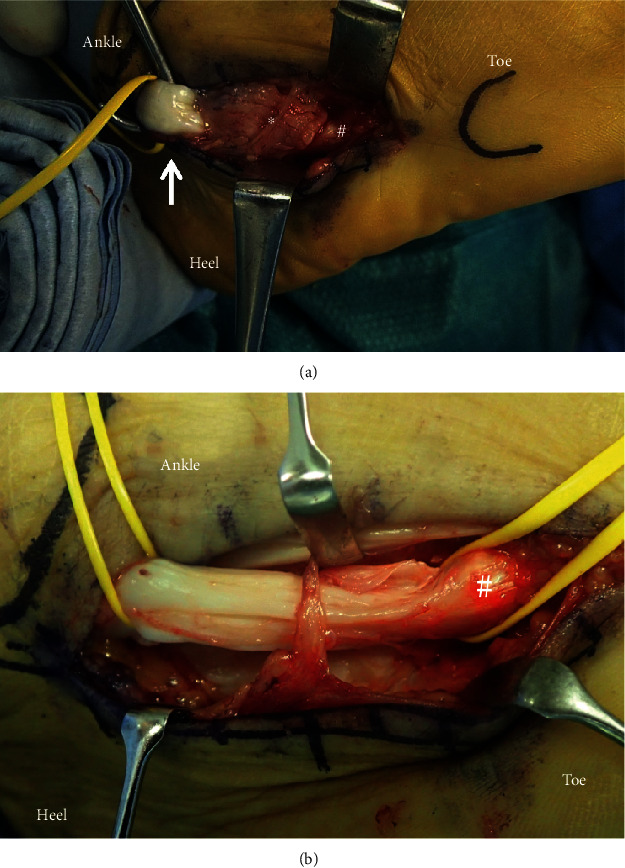
Intraoperative photograph: (a) peroneus longus tendon runs through hypertrophic peroneal tubercle. A traction of peroneus longus tendon causes hypertrophic peroneal tubercle and os peroneum to collide with each other. (b) Resection of hypertrophic peroneal tubercle released the impingement between os peroneum. ^∗^Hypertrophic peroneal tubercle, ^#^os peroneum.

**Figure 6:**
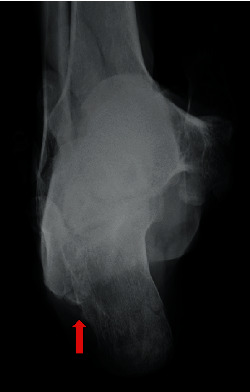
The patient's right foot one year after the procedure: the relapse of resected hypertrophic peroneal tubercle could not be found.

A lateral incision was made parallel to the peroneus longus tendon. During operation, we observed that traction of the peroneus longus tendon caused the HPT and OP to collide with each other (Figure 5). There was no apparent fracture or diastases of OP during inspection. The protuberance of the HPT was resected with a chisel. The resection of HPT released OP to glide smoothly along the peroneal tendon track. A 3 cm longitudinal tear of the peroneus longus tendon between the HPT and OP was confirmed and sutured with 3-0 PDS by using tubularization. The wound was irrigated and closed, and a sterile dressing was applied. Full weight-bearing was allowed postoperatively, as tolerated. The postoperative course was uneventful, and the patient returned to normal activities without any kind of functional disability. We compared the outcomes of the surgery, using the Japanese Society for Surgery of the Foot (JSSF) scale, an objective standard rating system [4, 5], and the Self-Administered Foot Evaluation Questionnaire (SAFE-Q) [6]. The SAFE-Q is an ankle-specific subjective evaluation method consisting of six subcategories (i.e., pain and pain-related, physical functioning and daily living, social functioning, shoe–related, general health and well-being, and sports activity [optional]). The preoperative JSSF score of 71 points (maximum score of 100 points) significantly improved to 100 points after one year. Compared to the preoperative condition, all subscale scores in the SAFE-Q improved after 1 year: pain and pain-related, 48 to 72 points; physical functioning and daily living, 41 to 82 points; social functioning, 62 to 88 points; shoe-related, 50 to 74 points; and general health and well–being, 58 to 76 points. Plain radiographs taken one year after the surgery showed no signs of HPT recurrence (Figure 6).

## 3. Discussion

The peroneal tubercle is a bony process at the lateral wall of the calcaneus, which is located between the peroneus longus tendon and peroneus brevis tendon. HPT is an enlargement of the peroneal tubercle, which in 20.5%-24% cases [7, 8] may occur owing to congenital qualities, trauma, in response to physical activity, and inflammatory changes related to the peroneus longus spasm [1, 9]. HPT could potentially cause stenosing tenosynovitis of the peroneus longus tendon, with HPT resection particularly effective for recalcitrant painful conditions [1, 10].

OP is a sesamoid bone located at the lateral aspect of the calcaneocuboid joint within the peroneus longus tendon [2, 11]. OP is reported to exist in 4%-29% of the population [12–15]. Its histologic structure is ossification, composed of fibrous tissue, cartilage, and bone [16]. Various foot pain caused by OP are defined as POPS, which results from fractures or diastases of multipartite os peroneum and damage in the peroneus longus tendon [17]. MRI images of POPS show edema of the bone marrow of OP and cuboid, as well as inflammatory changes of soft tissues [18].

In the present case, the HPT and OP coexisted on the affected side of the foot, making the determination of the exact cause of the lateral foot pain challenging at first instance. We used preoperative MRI and dynamic ultrasonographic images as a diagnostic tool. MRI revealed a localized bone marrow lesion on the HPT, but not on OP. These findings lowered the possibility of POPS, which shows edema of the bone marrow of OP and cuboid. Additionally, the dynamic ultrasonographic image of collision between the HPT and OP, and the release of pain with localized lidocaine injection between the HPT and OP allowed us to convince the cause of the pain as an impingement of HPT and OP. With these findings, we only addressed HPT resection without OP removal and achieved a successful result. From the literature review, we found that five cases with HPT coexisted with OP. All patients were treated with resection of both HTP and OP [2, 19]. Stockton reported four cases of HPT and OP resection followed by peroneus longus tenodesis to peroneus brevis. They mentioned that all had either enlarged OP or fracture. Pierson reported one case of HPT resection with OP enucleation, and the defect within the peroneus longus tendon was closed. Both studies did not clearly mention the rationale for resecting OP together with HPT. To the best of our knowledge, this is the first case report of HPT coexisting with OP surgically treated with only HPT resection without OP removal. OP resection potentially requires tenodesis of the peroneus longus tendon to the peroneus brevis tendon or tendon graft to restore its function, particularly when OP is too large to preserve the continuity of the peroneal longus tendon on its removal [20, 21]. Recovery can require a long duration, with 4-6 weeks of limited weight-bearing [21]. In the present case, the patient was able to achieve a quick recovery to a regular living with minimum physical or social restriction owing to being free from OP resection. Resection of OP should be discussed with extra caution for the cases of HPT coexisted with OP. In conclusion, we experienced the case of a patient with HPT coexisting with OP, successfully treated by resecting only HPT and retaining OP. Preoperative MRI and dynamic ultrasonographic imaging could be effective tools for identifying specific pain lesions.

## Data Availability

The data used to support the findings of this study are included within the article.
